# Antitumor Activity of Pt(II), Ru(III) and Cu(II) Complexes

**DOI:** 10.3390/molecules25153492

**Published:** 2020-07-31

**Authors:** Katarzyna Gałczyńska, Zuzanna Drulis-Kawa, Michał Arabski

**Affiliations:** 1 Institute of Biology, Jan Kochanowski University, Uniwersytecka 7, 25-406 Kielce, Poland; kgalczynska@ujk.edu.pl; 2 Department of Pathogen Biology and Immunology, Institute of Genetics and Microbiology, University of Wroclaw, Przybyszewskiego 63-77, 51-148 Wrocław, Poland; zuzanna.drulis-kawa@uwr.edu.pl

**Keywords:** cobalt, copper, ruthenium, imidazole, apoptosis, antitumor activity

## Abstract

Metal complexes are currently potential therapeutic compounds. The acquisition of resistance by cancer cells or the effective elimination of cancer-affected cells necessitates a constant search for chemical compounds with specific biological activities. One alternative option is the transition metal complexes having potential as antitumor agents. Here, we present the current knowledge about the application of transition metal complexes bearing nickel(II), cobalt(II), copper(II), ruthenium(III), and ruthenium(IV). The cytotoxic properties of the above complexes causing apoptosis, autophagy, DNA damage, and cell cycle inhibition are described in this review.

## 1. Introduction

The discovery of an antitumor effect of cisplatin (transition metal) led to the creation of the field of metal-based chemotherapeutics [1]. Platinum-based drugs such as cisplatin, oxaliplatin, and carboplatin are routinely used alone or in combination with other agents, to treat various malignancies such as testicular, lung, ovarian, colorectal, head and neck cancers [2,3,4,5,6,7]. Cisplatin binds to DNA, blocking the transcription and replication, which initiates the apoptosis process [8,9,10,11]. Cisplatin application is nevertheless limited due to an increasing resistance as well as due the side-effects associated with its toxicity [12,13,14]. Therefore, several non-platinum metallopharmaceuticals such as ruthenium(III)-, gold(I)-, gallium(III)-, copper(II)-, cobalt(II)- and nickel(II)-based compounds have been investigated for their anticancer potential [15,16,17,18,19,20,21,22,23]. Some of the ruthenium(III) complexes (NAMI-A and KP1019) are currently being tested in clinical trials [17]. The cytotoxic properties of metal complexes are widely studied; therefore, this review aims to gather this knowledge, with particular emphasis on the proposed mode of action. The main types of cell death induced by metal-based anticancer compounds are apoptosis and autophagy. Phenotypic changes associated with cell death may vary depending on the stimuli and cell type [24,25,26]. 

## 2. The Role of Metal Complexes in Apoptosis Generation

**Figure 1:**
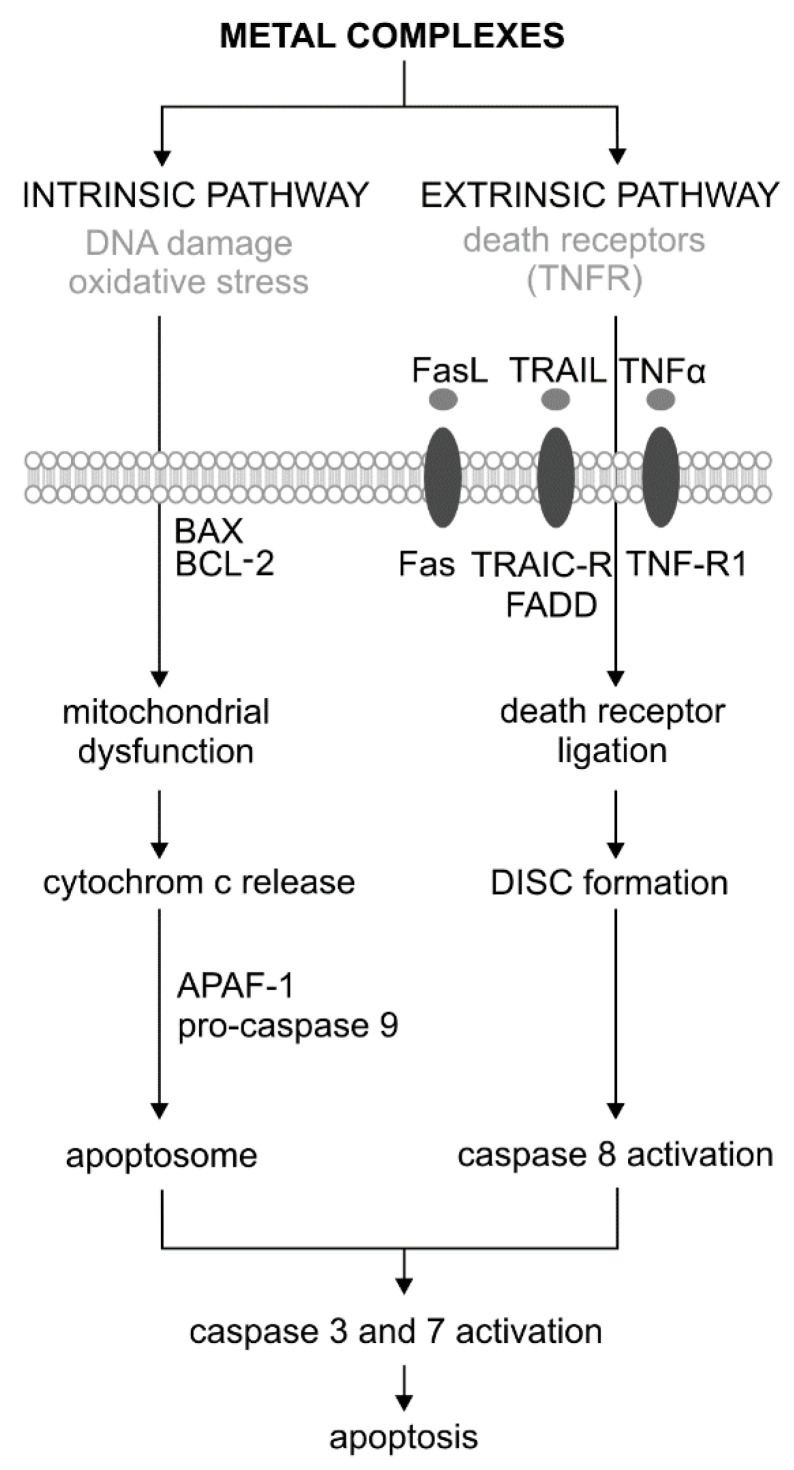
Extrinsic and intrinsic pathways of apoptosis and its activation by the metal complexes adopted [29]. DISC—Death-Inducing Signaling Complex.

Apoptosis is the first genetically identified programmed cell death process that can be induced by both external and internal factors. There are two main pathways of apoptosis: extrinsic (associated with cell death receptors) and intrinsic (mitochondrial) (Figure 1) [27,28]. During apoptosis, the initial symptoms are observed at the nucleus level, where chromatin condenses and is located just below the cell membrane. As a result, it comes to condensation and nucleus fragmentation. The next step is the condensation of cytoplasm and forming vesicles to the cell surface. As a result of their separation, the apoptotic bodies are formed. They include concentrated chromatin, cytoplasm, and cell organelles. In the end, apoptotic bodies are phagocytized by macrophages. This process involves the degradation of cellular components by a group of cysteine proteases called caspases. Caspases, by their structure and function, are divided into initiators (caspase −2, −8, −9 and −10) and effectors (executive) (caspase −3, −6 and −7). In response to the stress factor, the initiator caspases are activated in an intrinsic pathway through the apoptosome, or extrinsically formed by the DISC signaling complex (death-inducing signaling complex). Effector caspases are activated as a result of digestion by initiator caspases. The activation of the caspase cascade causes the proteolysis of important cellular proteins, including the DFF40/CAD endonuclease inhibitor—ICAD proteins (inhibitor of caspase-activated DNase). As a result of the proteolysis of nuclear lamine (the fibrillar protein that performs structural and regulatory functions in the cell nucleus during mitosis), the nucleus shrinks and fragments. In turn, they activate proteolytically active DNA endonuclease. As a result of the cytoskeleton protein (actin) digestion, the cell breaks down into apoptotic bodies (Figure 1). Effector caspases, mainly caspase 3, are also activated by proteins associated with the DNA damage pathway—DDR (DNA Damage Response). These include PARP (poly (ADP-ribose) polymerase), DNA-PKcs, and serine/threonine protein kinase ATM. An important protein in the process of apoptosis is also p53, which is involved in the various stages of activation of the extrinsic and intrinsic pathways. This protein is a transcription factor for proapoptotic genes and also blocks the action of anti-apoptotic proteins. It also stimulates many types of non-coding microRNAs, which in turn silence the proteins associated with the cell cycle and DNA repair [27,28].

The extrinsic (receptor) path of apoptosis is initiated by nutrient deficiency, as well as a local increase in the hormone and cytokine levels. Besides, the activating agents for the apoptosis receptor pathway are chemical compounds, such as cytostatic and physical factors, e.g., various types of radiation and temperature. The extrinsic pathway of apoptosis occurs via specific transmembrane receptors belonging to the tumor necrosis factor receptor (TNFR) superfamily. In addition to CD95/FAS and TRAILR (receptor of TNF-related apoptosis-inducing ligand), TNFR1 is also one of them. Ligand binding, e.g., Fas (type II transmembrane protein belonging to the TNF family), TNFR (tumor necrosis factor receptor), and tumor necrosis factor ligand TRAIL with death receptors, cause the oligomerization of the cell surface receptor and initiates the apoptotic cascade. The binding of the FADD adapter protein to Fas initiates the process of apoptosis by forming a signaling complex (DISC). The FADD–Fas complex also causes autoproteolysis and the activation of caspase 8, which initiates the extrinsic pathway of apoptosis (Figure 1) [30,31].

**Figure 2:**
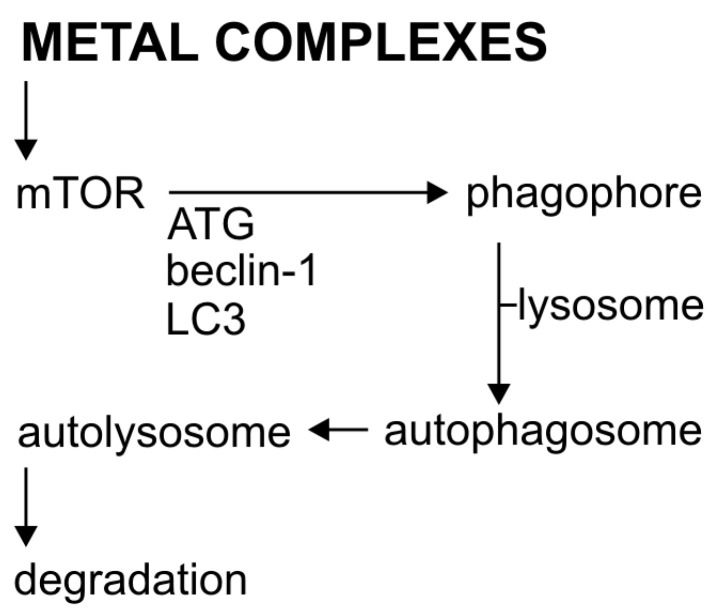
The process of autophagy and its activation by metal complexes.

The intrinsic pathway is initiated by cellular stimuli, e.g., DNA damage and oxidative stress. These stimuli cause mitochondrial dysfunction, including changes in the inner membrane, channel opening, and the loss of mitochondrial membrane potential [32]. Proapoptotic proteins, such as cytochrome c, are released from the mitochondrial intermembrane space into the cytosol. Cytochrome c binds and activates the apoptotic protease-activating factor 1 (APAF-1) as well as procaspase 9, forming an apoptosome. As a result of these changes, caspase 9 is activated [33]. The regulation of the release of mitochondrial factors, including cytochrome c, depends on the proteins from the BCL−2 family (Figure 2). This family consists of a large and diverse group of globular proteins. To date, 25 genes have been identified from this family. Most BCL−2 homologues are anti-apoptotic proteins, such as BCL−2, BCL-X, BCL-XL, BCL-XS, BCL-W, and BAG. In contrast, proapoptotic proteins include BCL−10, BAX, BAK, BID, BAD, BIM, and BIK [34].

The antitumor properties associated with apoptosis show, among others, two ruthenium complexes [imiH][*trans*-[Ru(*N*-imi)(*S*-dmso-S)Cl_4_] (imi = imidazole, dmso = dimethylsulfoxide), named NAMI-A, and [indH][*trans*-[Ru(*N*-ind)_2_Cl_4_] (ind = indazole), named KP1019, qualified for clinical studies [17]. Arena ruthenium(II) complexes binding to DNA have great potential as anticancer drugs [35]. The complex [(η^6^-aren)Ru(*N,N*-en)Cl]^+^, where en = 1,2-diaminoethane, aren = para-terphenyl, developed by Sadler’s research group, induces apoptosis by the inhibition of DNA synthesis, the activation of p53 protein, the downregulation of p21 and BAX gene expression, and nuclear fragmentation [36]. Organometallic ruthenium(II) compounds of the Ru(II)-PTA type exhibit promising antitumor properties. Ruthenium complexes from the above group, e.g., the RAPTA-C complex Ru(*ƞ^6^*- cymene)(PTA)Cl_2_, PTA = (1,3,5-triaza-7-phosphatricyclo [3.3.1.1]decane) induce apoptosis against Ehrlich ascites cancer by the mitochondrial pathway. This causes the changes in BAX to BCL-2 ratio, the release of cytochrome c, and the activation of caspase 9 [37,38,39,40,41]. 

Further examples of compounds widely used in medicine are ^67^Ga and ^68^Ga compounds. Gallium nitrate has anticancer properties and is used in a therapy under the trade name Ganitet. The mode of action of this compound is associated with the induction of apoptosis via the mitochondrial pathway [42]. Some gallium(III)-containing pyridine complexes have a higher antitumor activity than cisplatin. The activation mechanism of the [Ga^III^(LI_2_)_2_]ClO_4_ complex (where LI_2_ˉ is a deprotonated ligand with a 4,6-diiodophenol pyridine molecule) is associated with the inhibition of proteasome activity and the induction of apoptosis [43,44]. Similarly, Ga(III) complexes with thiosemicarbazone, show a 20 times stronger activity compared to commonly used cisplatin. The Ga(III) complex with thiosemicarbazone induces a p53-dependent and -independent apoptosis [45]. Cisplatin is also a compound that induces cell death by apoptosis, which causes DNA damage. It also induces autophagy as a cytoprotective response [46].

### 2.1. The Role of Metal Complexes in the Autophagy Process

Autophagy is a tightly regulated, basic catabolic process [47], in which the damaged cytoplasmic material and organelles are initially captured by autophagosomes, then sequentially combined with lysosomes to form autolysosomes (Figure 2) [48]. Autophagosome formation regulates the conserved autophagy-related proteins (Atg proteins). Currently, over 30 genes have been identified for these proteins [49] with those coding Beclin-1 (also called ATG-6) and LC3 (ATG-8), the two basic elements of cell autophagy [50,51]. The regulation of autophagy is quite complex. Important regulators of autophagy, both in normal and cancer cells, are mTOR kinase (the so-called mammalian target of rapamycin) and AMPK protein kinase. The mTOR plays a key role in transmitting autophagic stimuli [52]. AMPK protein kinase is the main indicator of metabolism that regulates lipid, cholesterol, and glucose metabolism [53]. The decrease in intracellular ATP production activates AMPK, which in turn inhibits the action of mTOR, thus causing autophagy [54]. Protein kinase B/Akt, which is an important effector for Class I phosphoinositide 3-kinase 2class III PI3K induces autophagy [55,56].

Autophagy, as previously mentioned, is a cytoprotective process antagonizing oxaliplatin-induced apoptosis. Unlike apoptosis, autophagy is not uniquely identified with the state of cell death. It is a mechanism activated in response to stress conditions, which results in the degradation of cytoplasmic proteins (damaged or unnecessary) or the elimination of whole organelles. Autophagy can function as a strategy of cell survival in the conditions of the limited availability of nutrients or stress. Although the exact role of autophagy in cell survival compared to death is highly context-dependent, increasing evidence indicates that autophagy may promote tumor cell survival in response to both cytotoxic and targeted chemotherapy [57,58]. The oxaliplatin treatment of hepatocellular carcinoma cells stimulated the autophagic response both in vitro and in vivo. On the other hand, the suppression of autophagy with pharmacological inhibitors (3-methyloadenine or chloroquine) and the RNA interference of important autophagic genes increased hepatocellular carcinoma death [59]. Similar results were obtained for the oxaliplatin treatment of gastric cancer cells MGC-803 [60]. In contrast, an oxaliplatin derivative E-Platinum-induced autophagy in BGC-823 gastric gland cancer cells, by suppressing the mTOR signaling pathway [61]. Autophagy was proven to be a mechanism that stimulates the death of cancer cells because its inhibition by the 3-methyloadenine or chloroquine causes cancer cell proliferation [61]. Brasseur et al. [62,63,64,65] described a panel of platinum(II) complexes tested on dependent- (ER^+^) and estrogen-independent (ER^-^) cells. The platinum(II) complex with 17-β-estradiol marked VP-128 showed a selective antiproliferative activity to hormone-dependent breast cancer cells. The mechanisms of this compound activity on ER-positive and ER-negative tumor cells turned out to be different [62,63,64,65]. It induced the activation of caspase 9/3 and the cleavage of PARP (poly-ADP-ribose) in ER^+^ cells, whereas, in ERˉ cells, the caspase-independent apoptosis was observed where the compound VP-128 induced the translocation of the proapoptotic factor (AIF) to cell nucleus [66]. 

### 2.2. Inhibition of Proteasome Activity by Metal Complexes

**Figure 3:**
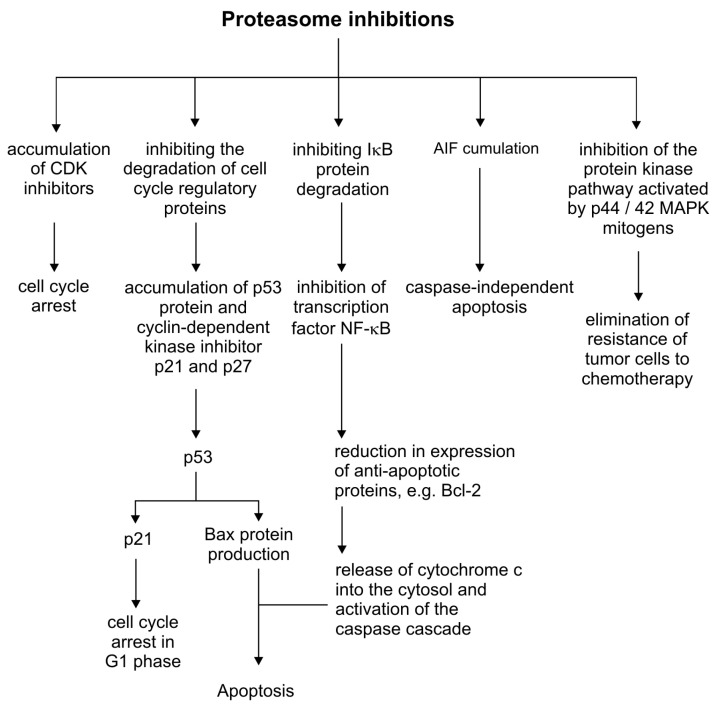
Inhibition of the proteasome in the regulation of cellular processes.

The proteasome is a multi-enzymatic complex that plays an important role in regulating cellular processes and cell proliferation. The human proteasome is named to as 26S and consists of a 20S core (also referred to as the 20S proteasome) and two 19S regulatory particles. It degrades the regulatory proteins, cyclin-dependent kinase inhibitors (e.g., p21 and p27), tumor suppressors (e.g., p53), and nuclear factor inhibitors (NF)-κB (e.g., IκB-α) that are necessary for tumor growth [67]. The potential therapy target of copper and its complexes could be the proteasome activity, by an ion-dependent inhibition. The disturbance in the proteasome regulation consequently will influence the degradation of cell cycle regulatory proteins, NF-ĸB pathway activation, the mitogen-activated protein kinase pathway (p44/42 MAPK) and the degradation of the apoptosis-inducing factor (AIF), altogether having a cytotoxic effect on cancer cells (Figure 3)[68].

An organic copper complex [Cu(8-OHQ)_2_] (8-OHQ - 8-hydroxyquinoline hemisulfate) showed the ability to inhibit chymotrypsin-like proteasome activity in cancer cells, which is associated with the induction of apoptosis [69]. Cu(II) and Zn(II) complexes with diethyldithiocarbamate ligand antitumor properties against MDAMB-23 cells inhibited the activity of the 26S proteasome in the cell more intensively than the purified core particle of the 20S proteasome itself. The authors suggested that this might be associated with the inhibition of the JAMM domain in the 19S proteasome cover. The 19S proteasome molecule is responsible for the recognition of ubiquitinated proteins and their further processing (cutting the ubiquitin chain) before degradation. The deubiquitinating activity of the 19S particle is dependent on a metalloisopeptidase with a coordinated zinc ion, and this structural motif in the 19S particle (JAMM domain) has been suggested as prospective target for anticancer drugs [70]. Gold(III) complex with dithiocarbamate, also showing antitumor properties against MDA-MB-231 breast cancer cells, inhibited the activity of the purified rabbit 20S and cellular 26S proteasome. This resulted in the accumulation of ubiquitinated proteins, the proteasome target protein p27 and the induction of apoptosis [71]. The copper complex with the pyrrolidine dithiocarbamate (PDTC) ligand was able to reduce the chymotrypsin-like activity of the proteasome, to suppress cell proliferation, to induce apoptotic cell death, and to inhibit the uptake of radiopharmaceutical 2-[^18^F]fluoro-2-deoxy-D-glucose in cultured human prostate cancer cells [72]. Similarly, the cobalt complex [Co^III^(L^1^)_2_ClO_4_] with the [NN′O] tridentate ligand HL1 inhibited the chymotrypsin-like activity of the proteasome in PC-3 cancer cells [73].

## 3. Interaction of Metal Complexes with DNA

Metal complexes can interact with DNA either directly or indirectly. Generated reactive oxygen species (ROS) in cells interact indirectly with DNA, where the hydroxyl radical is added to the double bonds of heterocyclic DNA bases and abstracting the hydrogen atom from the thymine methyl group, and each of the five 2’-deoxyribose carbon atoms causing DNA strand breaks and the formation of AP sites. In reaction with OH**˙,** the thymine radical and sugar radicals, as well as the OH**-**adduct with DNA bases are formed. The resulting radicals of thymine residues can react with oxygen to form peroxides. It leads to the formation of three isomers of thymidine peroxide that have a peroxide group in the 5-position or 6-position pyrimidine ring, or a carbon-bonded methyl group. The first two peroxides form a thymidine derivative called thymidine glycol. The third thymidine peroxide breaks down to form 5-hydroxymethyl-2’-deoxyuridine and 5-formyl-2’-deoxyuridine. Reactions of the hydroxyl radical with cytosine also cause the formation of derivative products analogous to thymine [74]. 

The hydroxyl radicals are also added to the purines giving rise to C4-OH-, C5-OH- and C8-OH-adduct radicals. The resulting adducts then dehydrate and oxidize purine radicals, which leads to the formation of 8-hydroxypurines (7,8-dihydro-8-oxopurine) and formamide pyrimidines, respectively. The addition of OH˙ to C8 guanine produces 8-hydroxyguanine and 2,6-diamino-4-hydroxy-5-formamido-pyrimidine. The analogous reaction of adenine forms 8-hydroxyadenine and 4,6-diamino-5-formamidopyrimidine [75].

The unique reaction of the C5-centered radical of the sugar in DNA is the addition to the C8 position of the purine ring in the same nucleoside, which leads to intramolecular cyclization, resulting in 8.5’cyclopurine-2’-deoxynucleosides [75]. 

DNA–protein bonds are also formed in free radical reactions. Thymine–tyrosine binding has been identified in mammalian chromatin in vitro and in cells exposed to free radical-generating systems. The DNA–protein cross-links are the result of the allyl thymine radical addition to the C3-position of the tyrosine ring in a protein placed near the DNA chain [75].

Hydroxyl radical generates multiple products in DNA, which generate various modifications of nitrogen bases and sugars, AP sites, thread breaks, and DNA–protein cross-links. The disruption of the cellular redox state can be initiated by an excessive ROS generation or by interfering with ROS metabolism [76]. The main compound that binds ROS in cells is glutathione (GSH). Lowering GSH levels and increasing ROS levels in cells are associated with the redox imbalance. 

The copper(II) complex with N-(2-hydroxyacetophenone)glycinate was proved to be a redox disruptor, reducing the intracellular GSH level due to the formation of water-soluble Cu–GSH conjugates. The treatment with the above compound caused the release of cytochrome c from the mitochondrial membrane, and activated the internal pathway of apoptosis [77]. Zhou et al. described the copper(II) complex with phenanthroline (phen) [Cu(phen)_2_], which induced the apoptosis in the Bel-7402 liver cancer cell line [78]. Cai et al. showed that the apoptosis pathway in the [Cu(phen)_2_]-treated cell line could be initiated by the accumulation of excessive copper, ROS generation, and the reduction in the GSH/GSSG (Glutathione disulfide) ratio [79]. This complex also exhibited strong cytotoxicity against human HL60 leukemia cells and SGC-7901 gastric cancer cells [80]. 

Metal complexes can also directly cause DNA damage. The most known anticancer compound that interacts with DNA is cisplatin. It is now widely accepted that the cisplatin mode of action is associated with DNA interaction. However, only a small amount of intracellular cisplatin was associated with genomic DNA (33%). The vast majority of the drug interacted with proteins or small molecules of the cell (e.g., glutathione) [81]. Cisplatin hydrolysis occurs in the cell and its cationic forms react with DNA to give numerous cisplatin–DNA adducts. It forms cross-links with 1,2-intrastrand between adjacent guanines d(GpG), between adjacent guanine and adenine d(ApG), and 1,3-intrands between purines separated by one or more bases d(GpNpG). Besides, inter-strand cross-links and DNA–protein cross-links may be formed. The most important interaction is 1,2-intrastrand d(GpG) binding to platinum, which is coordinated with adjacent N7 guanine atoms [82]. Platinum(II) complexes also can intercalate into DNA. A series of active complexes with antitumor properties of the type [Pt(IL)(AL)]^2+^ have an intercalating ligand (IL) and a non-intercalating ancillary ligand (AL). Complexes such as [Pt(phen)(en)]^2+^ intercalate with DNA minor groove, mainly between the base pairs C3-G4 and T2-A5, as a result of which the helix is lengthened and rigidified [83,84,85,86]. 

Metal coordination compounds can interact with DNA in several different ways. The simplest example is the [Pt(terpyridine)Cl]^+^ complex, initially intercalating with DNA, and losing the labile chloride ligand forms covalent bonds with nitrogen base pairs [83]. 

The ruthenium(III) complex marked with KP1019 prepared in Keppler laboratories interacts with DNA causing damage of different quantity and quality compared to cisplatin activity, but the exact mechanism is not yet elucidated. The ruthenium complex NAMI-A was also shown to interact with DNA *in vitro*, however, it is not critical for its anti-metastatic activity [82]. Intercalating metal complexes are also combined with N_4_-tetradentate ligands, such as combinations of copper(II), nickel(II) or zinc(II) complexes with the cation [M(*N,N’-bis*-5-(triethylammoniummethyl)-salicylidene-2,3-naphthalendiiminato)]^n+^. These complexes bind to DNA through the intercalation. It was observed that the mentioned nickel(II) complex with a square planar coordination geometry of the coordinating sphere, penetrates deeply between base pairs in DNA compared to other metal compounds. The copper(II) and zinc(II) complexes discussed above have an octahedral arrangement of donor atoms, which is probably the reason for the weaker interaction with DNA. 

The bis-(1,10-phenanthroline) copper(II) complex is well known for its ability to cleave DNA, especially in the presence of hydrogen peroxide [87]. The exact mechanism of action is still being studied, but it probably intercalates with DNA at the minor groove [88,89]. The DNA–copper complex is then oxidized in the presence of an activator, triggering the oxidetive stress pathways, which consequently leads to the hydrolysis of DNA–hydrogen bonds. Another copper(II) complex, [Cu(N9-ABS)(phen)_2_] (where N9-ABS = N-(9H-purin-6)-yl)benzenesulfonamine) in the presence of ascorbate intercalates with a DNA strand causing bond hydrolysis [90]. Copper(II) complexes, in which two phenanthroline ligands are linked by a serinol bridge at the 3 or 2 positions, showed an increased DNA affinity and nuclease activity [83,91].

Cobalt(III) complexes such as [Co(en)_3_]^3+^, [Co(en)_2_(bpy)]^3+^ and [Co(en)_2_(phen)]^3+^ (en = 1,2-diaminoethane, bpy = 2,2ʹ-bipyridine, phen = phenanthroline)) bind to DNA by groove mode resulting in hydrolysis. Zinc(II) and copper(II) dinuclear complexes with a cis or trans bridge azobenzene, bind within a minor DNA groove and can hydrolytically cleave the strand, but only in the cis form [83].

Zhang et al. [92] described the synthesis and characterization of 1-[3-(2-pyridyl)pyrazol-1-ylmethyl] naphthalene ligand and its octahedral complexes [M(L)_3_][ClO_4_]_2_ (M = Cu(II), Zn(II)) and the copper(II) complex showed a high affinity to DNA and cytotoxicity against human leukemia cell lines HL-60, gastric cancer BGC-823 and mammary tumor MDA-MB-435 [92]. The inhibition of the metabolic activity of the aforementioned cell lines was also caused by the [Cu(L)_2_(NO_3_)][NO_3_] complex based on the same ligand. Its activity was also confirmed against other cell lines, including prostate cancer PC-3M-1E8, hepatoma cells Bel-7402, and cervical cancer HeLa). The cytotoxic properties were associated with the ability to intercalate and generate DNA breaks [93]. 

## 4. Cell Cycle Inhibition by Metal Complexes

**Figure 4:**
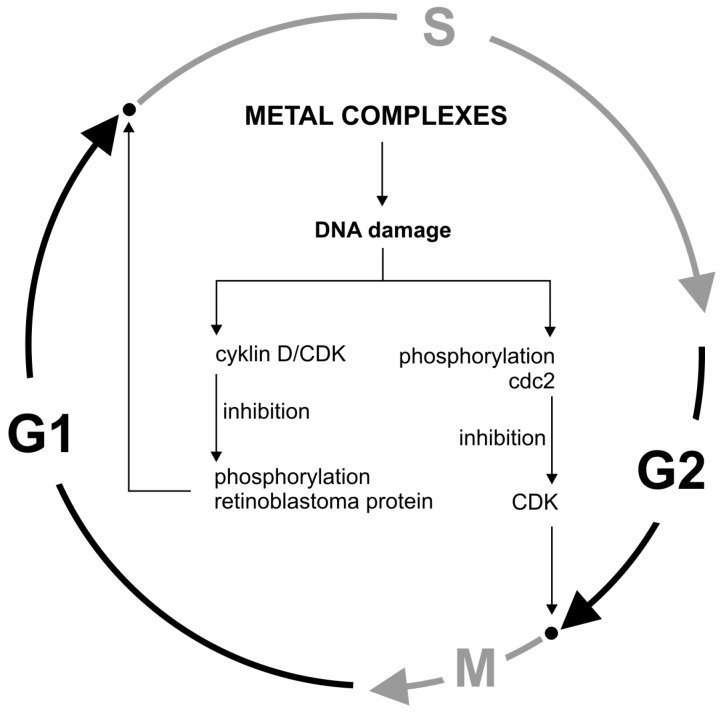
Cell cycle regulation by metals.

Stress inducers, including DNA-damaging agents, activate cell checkpoints functions, leading to cell cycle arrest. Cycle checkpoints existing in the G1/S, G2, and M phases have specialized systems to detect specific DNA structures indicating the damage or ongoing repair and replication process. An intracellular signal transduction cascade is initiated and the S phase is blocked. This is due to the inhibition of D/CDK cyclins that phosphorylate the retinoblastoma protein. Another way is to block the initial phase of mitosis by phosphorylating Cdc2 tyrosine and preventing CDK activation [94]. DNA-induced cell cycle arrest may consequently lead to cell death, e.g., by apoptosis (Figure 4). 

An example of a compound that causes cell death by inhibiting the cell cycle is the Pt(II) complex with terpyridine. It non-covalently interacts with DNA forwarded by the necrosis induction. This compound arrests the cell cycle in the G1 phase. It also induces a response to DNA damage by increasing the expression level of the γH_2_AX, p53, p21 genes, as well as phosphorylated kinase 2 (CHK2), an enzyme necessary for cell cycle arrest and DNA-induced apoptosis [95]. Some Ru(II) and Os(II) arenum complexes containing azopyridine or iminopyridine ligands stoped the cell cycle in the G0/G1 phase and induced the apoptosis through the caspase 3 activation [96]. Ruthenium(II) complexes containing carboline derivatives did the same but in the G2/M phase in HeLa cells, increasing ROS levels and mitochondrial damage [97]. Fluorescent Ru(II) complexes containing the HDAC group (histone deacetylases) caused the ROS generation, G0/G1 phase cycle arrest, and mitochondrial-mediated apoptosis [98]. The aforementioned RAPTA-C ruthenium complex inhibited the growth of Ehrlich ascites tumor cells in the G2/M phase accompanied with increased levels of p21 and a decreased expression of cyclin E [37,38,39,40]. Similarly, N-heterocyclic carbene Pd(II) complexes inhibit tumor cell proliferation by stopping the cell cycle in the G2/M phase and inducing apoptosis via the p53-dependent pathway [99]. In turn, the nitridoosmium(VI) complex, halt the cell cycle in S and G2/M phases killing HeLa cells [100]. Osmium(VI) complexes with pyrazole derivatives also show high cytotoxic effects in vitro through DNA damage, phase arrest S cell cycle, and apoptosis in HeLa cells [24,100]. Bolos et al. synthesized a copper(II) complex with a 2-amino-5-methylthiazole ligand, which was cytotoxic to human cells (cervical cancer HeLa, breast T47D, colon HT-29), while not affecting the normal mouse fibroblasts L-929. In cell lines treated with this compound, the inhibition of cell cycle progression as well as of DNA synthesis was observed, but not accompanied by the apoptosis process. The cell cycle arrest in the G2/M phase is probably associated with the inhibition of p34cdc2 kinase by the tyrosine phosphorylation and/or the induction of cyclin-dependent p21^WAF1^ kinase inhibitor [101]. Copper(II) complexes, [Cu(phen)(aa)(H_2_O)]NO_3_.xH_2_O (phen = 1,10-phenanthroline; aa = gly or DL-ala, sar, C-dmg) caused higher cytotoxicity in cisplatin-resistant MDA-MB-231 breast cancer cells than MCF10A. These complexes induced the apoptosis, cell cycle arrest, ROS generation, and double-stranded DNA breaks [102].

## 5. Conclusion

This article presents the main mechanisms of the in vitro anticancer activity of metal complexes (apoptosis, autophagy, cell cycle inhibition, DNA damage) described in the literature to date. Studies on cell lines indicate that metal complexes, especially copper complexes, are selective for cancer cells [103,104,105]. Metal complexes might soon prove to be good alternatives to cisplatin, which is still the most popular, but with many side effects and fast emerging drug-resistance in cancer cells. Metal complexes are an interesting option in the diagnostics and therapy of cancer. Due to their structure and properties, many of these compounds additionally have a high antitumor, anti-inflammatory, and antibacterial activity. Copper(II) complexes are very promising as an antitumor agent because their action is directed at copper levels and increases only in the cancer cell, with potential for proteasome inhibition and induction of apoptosis.

Due to intensive research work, metal complex compounds, as shown in the present article, show great application potential and may soon be used as anticancer drugs.
